# Simulated practice in the development of clinical reasoning in nursing students: A systematic review protocol

**DOI:** 10.1016/j.mex.2024.103144

**Published:** 2024-12-28

**Authors:** Rute Pires, Maria Marques, Henrique Oliveira, Margarida Goes, Miguel Pedrosa, Manuel Lopes

**Affiliations:** a Unidade Local de Saúde do Alentejo Central, Programa de Prevenção e Controlo de Infeções e Resistência aos Antimicrobianos, Évora 7000-811, Portugal; b Departamento de Enfermagem, Universidade de Évora, Évora 7000-811, Portugal; c Comprehensive Health Research Centre (CHRC), Universidade de Évora, Évora 7000-811, Portugal; d Instituto de Telecomunicações, Universidade de Aveiro, Aveiro 380-193, Portugal; e Universidade Católica Portuguesa, Lisboa, Portugal; f Unidade Local de Saúde do Alentejo Central, Gabinete de Formação, Investigação e Ensino em Enfermagem, Évora 7000-811, Portugal

**Keywords:** Simulation, Nursing students, Nursing education, Clinical reasoning, Pedagogical strategies, Clinical competence, Evidence-based education

## Abstract

*Simulated clinical practice* is a pedagogical technique that replicates real-world scenarios in a controlled environment, enabling nursing students to engage in the teaching-learning process actively. While simulated practice is a growing pedagogical strategy, several studies have examined its strengths and limitations. However, evidence of its effectiveness in developing clinical reasoning skills among nursing students still needs to be improved. This systematic review aims to assess the benefits of simulated practice in enhancing the clinical reasoning skills of undergraduate nursing students. Methods: A systematic review will be conducted using three databases: CINAHL, MEDLINE, and PubMed. The search strategy will include MeSH terms "simulation," "nursing students," "nursing education," and "clinical reasoning." Inclusion criteria: Studies published within the last five years (2017–2022) involving undergraduate nursing students and using simulated practice as an intervention. Two independent reviewers will conduct Data extraction and synthesis, with disagreements resolved by a third reviewer, as follows:

• Identify the benefits of simulated practice in clinical reasoning among nursing students.
• Analyze studies that utilize simulated practice as an intervention.
• Evaluate the effectiveness of simulated practice in developing clinical reasoning skills.

## Graphical abstract

This graphical abstract visually represents the core focus of the study, showcasing the interrelation between Nursing Students, Simulation, and Clinical Reasoning, along with associated pedagogical strategies and competencies.

**Figure:**
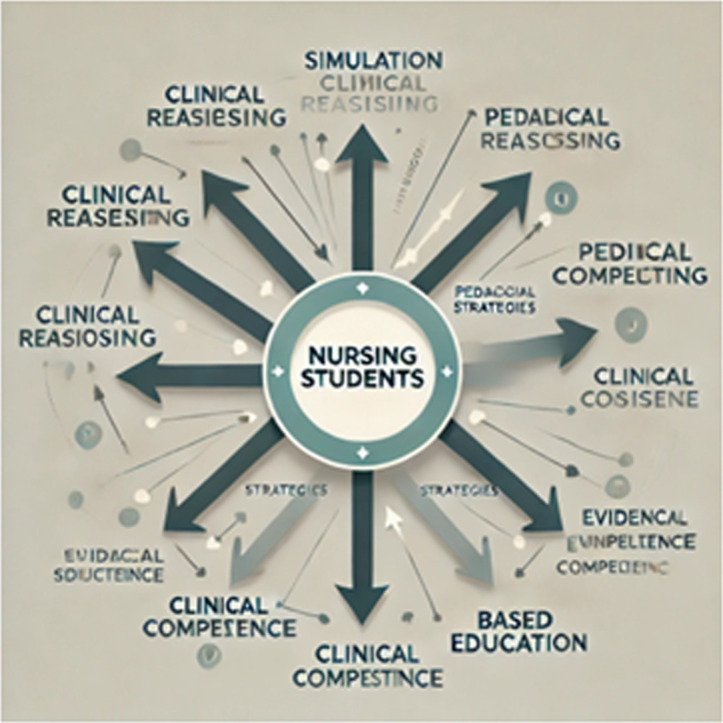

Specifications table

**Table:** 

Subject area:	Psychology
More specific subject area:	Clinical Reasoning in Nursing
Name of your protocol:	Simulated Practice in the Development of Clinical Reasoning in Nursing Students: A Systematic Review Protocol
Reagents/tools:	N/A
Experimental design:	This systematic review aims to assess the benefits of simulated practice in enhancing the clinical reasoning skills of undergraduate nursing students.
Trial registration:	PROSPERO registration number: CRD42022303916
Ethics:	This study is a systematic review, and as such, it does not involve direct research with human or animal subjects, nor does it involve data collection from social media platforms. Therefore, no ethical approval or informed consent is required. All data analyzed were sourced from previously published studies, which had already undergone the appropriate ethical review processes.
Value of the Protocol:	- Identifies the benefits of simulated practice in developing clinical reasoning among nursing students. - Provides a robust methodology for evaluating the effectiveness of simulated practice interventions in nursing education. - Establishes evidence to support the integration of simulated practice as a pedagogical strategy in nursing curricula.

## Background

Health care has become increasingly complex and demanding, requiring professionals to continuously enhance their skills, particularly their cognitive abilities. As both a discipline and a profession, nursing has evolved to address these challenges by developing a robust body of knowledge. This evolution aims to equip nurses with the necessary tools to solve complex problems and make informed decisions, thus fostering clinical reasoning skills [[1], [2], [3]].

Clinical reasoning, a fundamental clinical practice component, refers to the cognitive processes involved in care delivery. Young et al. describe clinical reasoning as the backbone of the care process, encompassing the reasoning that healthcare professionals use to solve and manage clinical problems. As such, clinical reasoning is integral at every stage of the care process [[4], [5]].

However, challenges in clinical practice training persist, particularly regarding the gap between theory and practice. Nursing education should adopt constructivist-based strategies to address these issues, where knowledge is central. Simulation of clinical practice is an increasingly recognized pedagogical tool to bridge this gap [6]. In her research, Benner argued that providing students with diverse learning opportunities through simulated clinical scenarios, coupled with close supervision from instructors who encourage reflection during and after these scenarios, can significantly enhance the development of clinical reasoning skills before students engage in real-world environments [7].

*Simulated practice* is a pedagogical strategy designed to develop nursing students' knowledge and skills. It involves recreating clinical environments through controlled yet realistic scenarios. This methodology, widely adopted by institutions and educators, aims to improve students' access to clinical skills training, with a strong focus on excellence in patient care. However, achieving such outcomes requires robust scientific evidence demonstrating the benefits of simulation—not only in the transfer of knowledge but also in developing skills and attitudes applicable to real-world contexts [[8], [9], [10], [11]].

Despite the increasing use of simulation, existing scientific evidence, although limited, already establishes a significant correlation between simulation and the development of critical thinking, and by extension, clinical reasoning, in nursing students. Most existing studies focus on the realism of simulators and scenarios, students' satisfaction with simulated experiences, self-efficacy, confidence, communication, motivation, skill transfer to clinical practice, and reflection-in-action.

Although simulation is widely accepted within the educational community, it remains a relatively recent strategy with limited evidence. Additionally, student participation in previous studies has been inconsistent, potentially influenced by their perceptions of simulation and simulators, which may introduce bias into the findings. Therefore, this review aims to identify the benefits of simulated practice in developing clinical reasoning in nursing students, addressing the following research question: Is simulation effective in developing clinical reasoning in nursing students?

## Description of protocol

### Materials and methods

This systematic review protocol was developed in accordance with the PRISMA guidelines (Preferred Reporting Items for Systematic Reviews and Meta-Analyses), which provide a structured framework for reporting the article selection process through a flow diagram that details each step until the final sample is obtained. The protocol is registered in the Prospective International Register of Systematic Reviews (PROSPERO) under the registration number CRD42022303916. Initially developed in February 2022, the protocol underwent amendments in September 2022. The completion of the systematic review is projected for the end of February 2023.

### Eligibility criteria

• Population

Inclusion criteria: Studies assessing the impact of simulated practice on the development of clinical reasoning in undergraduate nursing students in comparison with traditional teaching methodologies published between January 2017 and September 2022 in English, Portuguese, and Spanish. Studies involving nursing students at other educational levels or those that do not include simulated practice as an intervention will be excluded.

• Intervention

The review will include studies focusing on interventions based on simulated practice for undergraduate nursing students, regardless of geographical location. The primary goal of these interventions is to enhance clinical reasoning skills, ultimately improving the quality of care provided.

• Comparison

This systematic review will prioritize studies that include a comparison group to evaluate the effectiveness of simulated practice.

• Primary Outcome

The primary outcome of this review is to determine whether simulated practice enhances the clinical reasoning level among undergraduate nursing students. The review will prioritize studies that assess clinical reasoning using validated instruments aligned with the four domains of the Clinical Judgement Model.

• Study Design

This systematic review will preferentially include quantitative studies to ensure a comprehensive assessment of the evidence.

• Context

The review will include studies focusing on simulated practice interventions designed for nursing students within a teaching-learning context to improve educational outcomes.

### Search strategy

The search strategy will involve conducting a comprehensive bibliographic search across the following databases: CINAHL Plus with Full Text, MEDLINE, and PubMed.

The search strategy will combine four key concepts from Medical Subject Headings (MeSH), using the Boolean operator "AND": "simulation," "nursing students," "nursing education," and "clinical reasoning." This strategy will be customized for each database.

The search strategy will be adjusted according to the specific operators and fields of each database. For example:

• In PubMed, MeSH terms will be combined with free-text keywords to maximize the retrieval of relevant studies.
• In CINAHL, specific subject headings unique to this database will be employed alongside keywords to refine the search results.
• In MEDLINE, Boolean operators will be used strategically to combine subject headings and text words, ensuring comprehensive coverage of the research question.

These adjustments will ensure the search strategy is optimized for each platform, capturing all relevant studies for inclusion in the review.

### Data collection and analysis

• Selection of Studies

The studies retrieved from each database will be exported to Mendeley, where duplicates will be removed. To minimize bias, two reviewers will independently evaluate the search results in two stages: (1) an initial screening of titles, abstracts, and keywords to determine eligibility based on the inclusion criteria; (2) a full-text review of all potentially eligible articles. In cases of disagreement or uncertainty, a third reviewer will be consulted to reach a consensus. The entire selection process will be presented in a PRISMA flowchart, depicting the article selection strategy up to the final sample.

• Data Extraction

During the data extraction process, a descriptive assessment of each study will be conducted to collect information relevant to the review question. The title, origin, authors, methodology, participants, objectives, interventions, results, conclusions, and/or limitations are included. Specific details regarding the interventions will also be extracted, such as type, duration, number of students involved, and the type of skills developed. Two reviewers will independently perform data extraction, with any disagreements or uncertainties resolved by consultation with a third reviewer.

• Quality Appraisal

The methodological quality of the studies will be assessed using the Joanna Briggs Institute (JBI) critical appraisal tool, which will be tailored to the type of study being analyzed. The assessment will focus on the results’ internal validity, reliability, and applicability. Two reviewers will independently perform data extraction, with any disagreements or uncertainties resolved by consultation with a third reviewer.

Each item in the JBI tool will be scored as “yes,” “no,” or “not applicable.” Studies will be required to achieve a minimum score of 70 % to be included in the review. The quality assessment results for each study will be presented in detail, ensuring transparency in the selection process. Only studies meeting the established quality standards will be included, thereby ensuring that the evidence produced in this review is robust and reliable.

This systematic and rigorous approach minimizes bias and enhances the validity of the findings synthesized in the review.

• Strategy for Data Synthesis

The extracted data will be synthesized to support valid and logical conclusions. Data synthesis will involve collecting, combining, and summarizing the results from the individual studies included in the review. After evaluating the quality of the studies, extracting relevant data, and drawing conclusions, sufficient evidence should be available to answer the research question posed by this systematic review conclusively. Results will be grouped into categories and subcategories based on the type and number of studies analyzed. A table will be created to present key study characteristics, such as author, year, population, objectives, methods, interventions, results, conclusions, and limitations, facilitating the analysis and discussion of the findings. Additionally, tables, graphs, and/or figures will be generated to visually present the synthesized data, making cross-study comparisons easier. All team members will participate in this process to ensure the clarity and accuracy of data presentation.

### Protocol validation

Clinical reasoning is a fundamental component of clinical competence; however, traditional teaching-learning models, still widely used in many institutions, often need to adequately support the development of the desired clinical reasoning skills [12,13]. The literature also highlights challenges in clinical practice training, particularly the persistent gap between theory and practice. While clinicians focus on decision-making based on their existing knowledge, academics aim to generate knowledge on optimal approaches to care across the human life cycle [6,14].

Therefore, nursing education must shift away from traditional models and incorporate strategies that actively foster the development of clinical reasoning. This approach places nursing students at the center of the educational process, encouraging them to take an active role in managing their learning autonomously and becoming key contributors to the development of their own skills. Current educational demands require the application of strategies that promote higher-level reasoning, both theoretically and practically [[15], [16], [17], [18], [19]].

Simulation and simulated practice have emerged as effective teaching techniques that replicate clinical scenarios in a controlled yet realistic environment. These methods allow students to engage in the teaching-learning process actively, providing opportunities to practice, learn, and reflect [20]. Simulation is undoubtedly a student-centered teaching approach, where students are immersed in clinical scenarios, performing nursing actions as they would in real environments. This experience helps smooth the transition to clinical practice when students encounter similar situations.

Simulated practice not only enhances students' interest and motivation for learning but also leads to greater satisfaction. It promotes the integration of cognition, critical thinking, reflective thinking, and pedagogical objectives—all of which are essential for the development of clinical reasoning and decision-making skills in nursing students [8,21,22].

This literature review aims to identify the benefits of simulation in developing students' clinical reasoning and to assess its overall effectiveness.

## Limitations

Although this systematic review aims to provide comprehensive evidence on the effectiveness of simulated practice, some limitations should be noted. The inclusion criteria restrict the review to studies published between 2017 and 2022, which may exclude relevant earlier research that could provide additional context. Furthermore, qualitative studies were excluded, which might limit the understanding of subjective experiences and contextual factors influencing the development of clinical reasoning. Despite these limitations, the review is expected to provide robust evidence to guide future educational strategies.

## CRediT authorship contribution statement

**Rute Pires:** Conceptualization, Methodology, Resources, Writing – original draft, Writing – review & editing. **Maria Marques:** Conceptualization, Writing – review & editing, Supervision. **Henrique Oliveira:** Conceptualization, Validation, Supervision. **Margarida Goes:** Methodology, Validation, Resources, Writing – review & editing. **Miguel Pedrosa:** Resources. **Manuel Lopes:** Conceptualization, Validation, Resources, Writing – review & editing, Supervision.

## Declaration of competing interest

The authors declare that they have no known competing financial interests or personal relationships that could have appeared to influence the work reported in this paper.

## Data Availability

Data will be made available on request.

## References

[1] Marques, F.M., José, M., and Vinheira, P. (2020). Estudante de enfermagem em ensino clínico: estudo qualitativo da tipologia de decisão. In L. Fornari, F. Freitas, E. S. F. de Oliveira, C. Oliveira, and A. P. Costa (Eds.), Investigação Qualitativa Em saúde: Avanços e desafios (Vol. 8, pp. 121–129). Ludomedia. Retrieved from https://ria.ua.pt/handle/10773/32634

[2] Marques M.F., David C.L., Santos M.A., Neves S.C., Pinheiro M.J., Leal M.T. (2021). Percepções dos estudantes finalistas em enfermagem sobre a tomada de decisão clínica. Rev. Bras. Enferm..

[3] Quaresma A., Xavier D.M., Cezar-Vaz M.R. (2019). Raciocínio clínico do enfermeiro: uma abordagem segundo a teoria do processo dual. Rev. Enferm. UERJ.

[4] Young M.E., Thomas A., Lubarsky S., Gordon D., Gruppen L.D., Rencic J., Ballard T., Holmboe E., da Silva A., Ratcliffe T., Schuwirth L., Dory V., Durning S.J. (2020). Mapping clinical reasoning literature across the health professions: a scoping review. BMC Med. Educ..

[5] Carvalho C., Oliveira-Kumakura D.S., Railka A., Ramalho C., Morais V. (2017). Raciocínio clínico em enfermagem: estratégias de ensino e instrumentos de avaliação. Rev. Bras. Enferm..

[6] Thofehrn M.B., Leopardi M.T., Amestoy S.C. (2008). Construtivismo: experiência metodológica em pesquisa na enfermagem. Acta Paulista de Enfermagem..

[7] Benner P. (1984).

[8] Mota L., Monteiro C., Pacheco C., Francisca M., Amador R., Oliveira T. (2019). Factors influencing simulated practice in nursing education: scoping review. Millenium.

[9] Lei Y.Y., Zhu L., Sa Y.T.R., Cui X.S. (2022). Effects of high-fidelity simulation teaching on nursing students’ knowledge, professional skills and clinical ability: a meta-analysis and systematic review. Nurse Educ. Pract..

[10] Padilha J.M., Machado P.P., Pereira A.R., Ramos J.C., Pinto R.A. (2019). Clinical virtual simulation in nursing education: randomized controlled trial. J. Med. Internet Res..

[11] Park S., Hur H.K., Chung C. (2022). Learning effects of virtual versus high-fidelity simulations in nursing students: a crossover comparison. BMC Nurs..

[12] Costa R.R.de O., Medeiros S.M.de, Martins J.C.A., Menezes R.M.P.de, Araújo M.S.de (2015). O uso da simulação no contexto da educação e formação em saúde e enfermagem: uma reflexão acadêmica. Espaço Para Saúde - Rev. Saúde Pública. do Paraná.

[13] van Wyngaarden A., Leech R., Coetzee I. (2019). Challenges nurse educators experience with development of student nurses’ clinical reasoning skills. Nurse Educ. Pract..

[14] Meleis A.I. (2012).

[15] Chiavaroli N. (2017). Knowing how we know: an epistemological rationale for the medical humanities. Med. Educ..

[16] Martins J.C.A., Mazzo A., Baptista R.C.N., Coutinho V.R.D., de Godoy S., Mendes I.A.C., Trevizan M.A. (2012). The simulated clinical experience in nursing education: a historical review. Acta Paulista de Enfermagem..

[17] Mohammadi-Shahboulaghi F., Khankeh H., HosseinZadeh T. (2021). Clinical reasoning in nursing students: a concept analysis. Nurs. Forum..

[18] Queirós P.J.P. (2014). Reflexões para uma epistemologia da enfermagem. Texto Contexto Enfermagem..

[19] Rillo A., Martínez-Carrillo B.E., Jaimes-García J., Elizalde-Valdés V.M. (2017). Campos problemáticos para un curso de epistemología de las ciencias de la salud. Humanid. Méd..

[20] Martins J. (2017). Learning and development in simulated practice environments. Rev. Enferm. Ref., IV Série.

[21] Duarte H.M.S., Dixe M.D.A.C.R. (2021). Clinical decision-making in nursing scale (CDMNS-PT©) in nursing students: translation and validation. Rev. Bras. Enferm..

[22] Nunes J.G.P., Amendoeira J.J.P., Cruz D.A.L.M., Lasater K., Morais S.C.R.V., Carvalho E.C. (2020). Clinical judgment and diagnostic reasoning of nursing students in clinical simulation. Rev. Bras. Enferm..

